# Strengthening Behavior of Cemented Paste Backfill Using Alkali-Activated Slag Binders and Bottom Ash Based on the Response Surface Method

**DOI:** 10.3390/ma13040855

**Published:** 2020-02-13

**Authors:** Qi Sun, Xueda Wei, Tianlong Li, Lu Zhang

**Affiliations:** School of Civil Engineering, Liaoning Technical University, Fuxin 123000, China; sunqi@lntu.edu.cn (Q.S.); 471820438@stu.lntu.edu.cn (T.L.); 471920516@stu.lntu.edu.cn (L.Z.)

**Keywords:** bottom ash, alkali-activated slag, response surface method, optimization, microstructure

## Abstract

A new type of cemented paste backfill (CPB) was prepared by using the bottom ash (BA) from a thermal power plant as an aggregate, alkali-activated slag as a binder, and an air-entraining agent as an admixture. Based on the central composite design (CCD) response surface method, the mix ratio was optimized, and scanning electron microscopy-energy dispersive spectroscopy (SEM-EDS) was performed on the optimal mix ratio. ImageJ software was utilized to determine the porosity of the experimental samples at various curing ages. The results indicate that the optimal mix ratio of the aggregate-binder ratio is 3.28, the alkali dosage is 3%, the solid content is 67.44%, and the air-entraining agent dosage is 0.1%. As the curing age increases, the porosity of CPB gradually decreases. A calcium aluminosilicate hydrate (C-A-S-H) gel is the main hydration product of alkali-activated slag. At the beginning of the hydration reaction, the slag gradually dissolves, and the C-A-S-H product binds the BA together. At 14 d, complete calcium hydroxide (CH) crystals appeared in the hydration product. Finally, the degree of C-A-S-H crystallization increased further to form a dense structure.

## 1. Introduction

Coal-fired thermal power plants produce a large amount of bottom ash (BA) [1,2,3,4,5,6]. BA accumulates in the soil and ponds near power plants. The toxic substances contained in the BA seriously pollute the soil and groundwater, posing a substantial threat to humans and the environment [5]. Therefore, BA must be treated and utilized, and it is useful to prepare BA as a cemented paste backfill (CPB) material for goaf filling. CPB is composed of aggregates, binders, water, and additives. Using BA as a CPB aggregate is extremely beneficial for urban development and environmental protection. Compared with ordinary Portland cement, alkali-activated slag has a low cost and good durability and can be used as a binder with great potential to replace cement. Paste backfill technology is important for converting solid waste into CPB for goaf filling, which can control mining subsidence and make full use of solid waste and mineral resources [7]. Furthermore, to obtain CPB with good workability, good mechanical properties, and low cost, mix ratio optimization can be performed through a response surface method.

In recent years, the by-products associated with the mining of mineral resources may not be sufficient to completely fill mined-out areas [8]. Many scholars have also begun to use a variety of other solid wastes. Wu et al. [8] mixed fly ash with aggregate and researched the drainage of coal gangue-fly ash backfill (CGFB) under different stresses and the evolution of strength under different curing conditions with different fly ash contents by using self-developed equipment. Chen et al. [9] prepared CPB using construction demolition waste (CDW) and phosphogypsum (PG) and studied the mass concentration, the amount of CDW, the ratio of PG to CDW, the ratio of aggregate-binder to slump, the setting time, and the unconfined compressive strength (UCS) along with microstructure analysis to verify the CPB performance while using a static leaching experiment to evaluate the CPB impact on the environment. Yılmaz et al. [10] used CDW to partially replace tailings and studied the effect of the CDW substitution rate on the mechanical properties, durability, and microstructure of CPB. Li et al. [11] prepared CPB with PG and discussed the fluidity, UCS, and chemical stability of CPB with different PG dosages when the mass concentration was 60–66%. The study showed that PG-based CPB could maintain the mine stability and that the leaching rate of toxic substances was within the safe range. Zhang et al. [12] addressed the problem of the poor mechanical properties of CPB caused by tailings sand that was too fine in a Gobi mining area. CPB was mixed with aeolian sand to discuss the strength, fluidity, and flow of different aeolian sand contents at different mass concentrations. The results show that the incorporation of aeolian sand can compensate for the lack of physical and chemical properties of tailings sand and can offer a new idea for economical filling material for the Gobi mining area. Deng et al. [13] used waste rock as coarse aggregate and fly ash as fine aggregate to prepare CPB and focused on the influence of the solid content and composition on the strength and rheological properties of the backfill.

Research regarding the new CPB binder includes a study by Jiao et al. [14], who used a variety of characterization methods such as X-ray diffraction (XRD), thermogravimetric-differential scanning calorimetry (TG-DSC), scanning electron microscopy (SEM), and energy dispersive spectroscopy (EDS) for experiments in which a single Ge slag was used as an admixture to partially replace cement, and different dosages of Ca(OH)_2_, AlCl_3_, NaAlO_2_, and Na_2_CO_3_ were used as activators to stimulate the activity of Ge slag and partially replace cement. Among these compounds, NaAlO_2_-activated Ge slag worked best, and the amount of NaAlO_2_ was optimized for activating Ge slag. It was finally determined that 0.6 wt% NaAlO_2_, 15 wt% Ge slag, and 85 wt% cement are the optimal amounts. Jiang [15] et al. used alkali-activated slag as a binder of CPB and explored the effects of different solid contents, amounts of binder, ratios of activator to binder, ratios of sodium silicate and sodium hydroxide in the activator, and curing temperature along with other factors on the workability and compressive strength of CPB. Sun et al. [16] used a fly ash geopolymer as a binder and studied the mixing ratio of CPB based on a combination of the response surface method and multi-objective optimization; they obtained the Pareto frontier considering the unconfined compressive strength (UCS) and slump and obtained the optimal mix ratio of CPB by using the desirability function. Ercikdi et al. [17] used industrial wastes (including waste glass, fly ash, blast furnace slag, and silica fume) with pozzolanic properties to partially replace cement to prepare CPB and studied the effects of pozzolanic substance incorporation on the early and long-term strength. The study found that CPB mixed with fly ash, blast furnace slag, and silica fume had a slower strength increase in the early stage than cement and a lower long-term strength loss than cement. Cihangir et al. [18] used water glass and sodium hydroxide to stimulate slag as a binder to prepare CPB and found that CPB with alkali-activated slag as a binder has better resistance to acid and sulfate attack than cement, so the CPB of alkali-activated slag as a binder is less affected by the sulfide erosion in tailings, without long-term strength loss, and its 56-d strength is much greater than the 360-d strength of CPB with the same cement dosage. 

The response surface method is a statistical-based technique for investigating the best response in a region where it may be applicable, or at least better understanding any response affected by multiple variables. Many scholars have used the response surface method as a tool for material mix design. Fall et al. [19] used the UCS, slump, solid concentration cost (based on cement cost) of the CPB as response values and optimized the CPB formulation using the response surface method. Bouzalakos et al. [20] prepared controlled low-strength materials (CLSM) using mixture design and response surface methods. The optimal mix ratio was obtained with minimum cement usage, maximum byproduct usage, and a UCS target value of 2 MPa. Ferdosian et al [21]. used the response surface method to optimize the mix ratio of ultra-high performance concrete (UHPC), in which the workability and compressive strength were the main performance indicators. The response surface method is an effective mix design method.

Many scholars have researched the preparation of CPB with solid waste as an aggregate and binder, but few reports have discussed the preparation of CPB with BA as the aggregate and alkali-activated slag as the binder based on the response surface method.

In this paper, BA is used as an aggregate, alkali-activated slag is used as a binder, and an air-entraining agent is used as an additive to prepare a new type of CPB. The aggregate-binder ratio (mass ratio of BA to slag), alkali dosage (sodium hydroxide as a percentage of slag mass), solid content (mixture solids as a percentage of total mass), and air-entraining agent dosage (air-entraining agent as a percentage of slag mass) were considered as variables. The response surface method of the central composite design (CCD) is used to establish the CPB material mix ratio design to obtain response surface models of the slump, 28-d UCS, and cost. Then, the overall desirability function method is used to optimize the multi-objective mix ratio. SEM and EDS were used to study the hydration products of CPB under the optimal mixing ratio and the microstructure evolution mechanism under different curing ages.

## 2. Materials and Methods

### 2.1. Materials

The raw materials used in this research include BA, slag, alkali activator (sodium hydroxide), an air-entraining agent, and mixing water.

**Figure 1:**
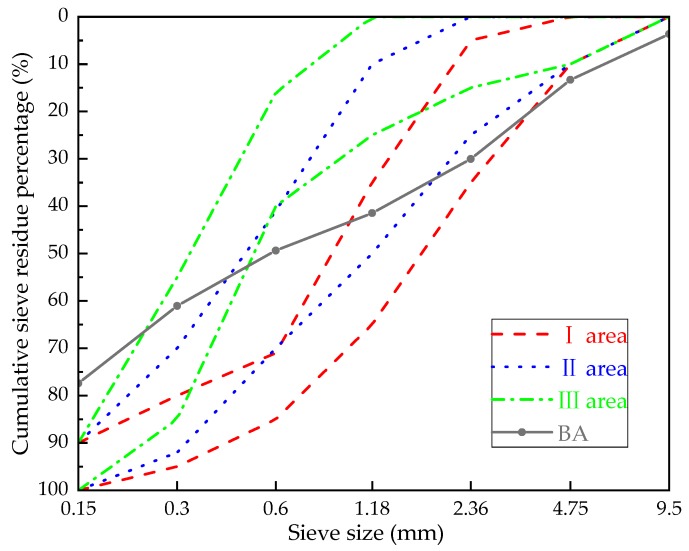
Screening curves of bottom ash (BA).

**Figure 2:**
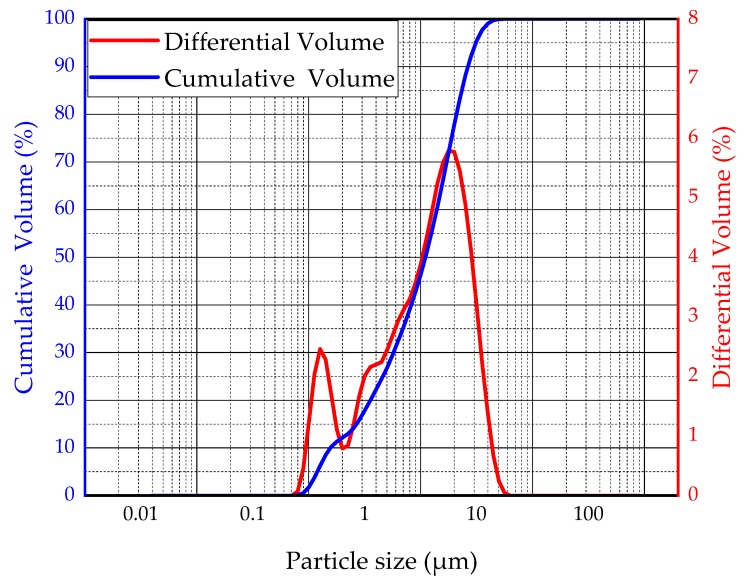
Particle size curve of slag.

(1) BA: The BA used in this research was taken from a power plant in Fuxin City, Liaoning Province, China. The main chemical composition obtained by X-ray fluorescence (XRF) analysis is shown in Table 1. According to the Chinese standard “Sand for building” (GB/T14684-2011) [22], the screening curves of BA are shown in Figure 1. The bulk density of BA is 801.7 kg/m^3^.
(2) Granulated blast furnace slag: The slag used in this research is Panlongshan brand S95 slag powder produced by Shandong Kangjing New Material Technology Co., Ltd, Shandong, China. The main chemical composition is shown in Table 1. The particle size distribution of slag was characterized by BT-2003 laser particle size analyzer (Dandong Bettersize Instrument Co., Ltd, Liaoning, China) as shown in Figure 2. The density of slag is 2920 kg/m^3^, and the specific surface area is 397.6 m^2^/kg.
(3) Alkali activator: The alkali activator used in this research is 96% analytical NaOH produced by Liaoning Quanrui Reagent Co., Ltd, Liaoning, China.
(4) Air-entraining agent: The air-entraining agent used in this research is the SY-5 air-entraining agent produced by the Jinan Shunxin Chemical Plant, and its main component is triterpenoid saponin.
(5) Mixing water: The mixing water used in this research was tap water.

### 2.2. Mix Design

The response surface method is a quadratic regression method that can build a functional model between response values and factors. Hence, the response surface method is a collection of statistical and mathematical techniques that is useful for developing, improving, and optimizing processes. The most practical response surface method is CCD. In this study, the CCD design method was used, with the aggregate-binder ratio, alkali dosage, solid content, and air-entraining agent dosage as variables, and the slump, 28-d UCS, and cost of CPB were used as the response values. Table 2 summarizes the factors, numbers, levels, and units of the CPB mix design under the CCD design method.

### 2.3. Experimental Methods

CPB is prepared according to the combination ratio of the CCD design method. First, the BA and slag are mixed for 2 min, and then the NaOH and the air-entraining agent are dissolved in the mixed water, poured into a mixer, and stirred for 2 min. The moisture content of the BA obtained from the on-site sampling is extremely large. The BA should be tested for moisture content before remixing and counted in the mixing water. The CPB mixture was used to fill a cylindrical mold of Φ 50 mm × 100 mm and was demolded after being cured for 24 h to obtain a cylindrical specimen. The test specimens were maintained under the standard curing conditions of a temperature of (20 ± 5) °C and a humidity above 95% in a SHBY-90B-type curing box.

In this study, a slump test of the CPB mixture was conducted according to the International Organization for Standardization(ISO) standard: “Testing of concrete Part 2: Properties of fresh concrete(ISO 1920-2)”. [24] According to the literature [16], the optimal slump of CPB mixtures is approximately 200 mm.

The upper and lower surfaces of the cylindrical CPB specimens were ground and subjected to a UCS test. The UCS of the CPB samples was tested in accordance with American Society for Testing and Materials(ASTM) standard: “Standard Method for Compressive Strength of Cylindrical Concrete Specimens (ASTM C 39/C39M-15a)” [25]. The load was applied at a loading speed of 1 mm/min until the specimen was broken. The tester records the complete stress–strain curves by plotting a data point every 0.01 kN, and then calculates its peak intensity. Five specimens were taken in each group for a parallel test, and the arithmetic mean was calculated (accurate to 0.01 MPa).

The procedure of the cost estimation method in this study is as follows: First, the apparent density of the CPB mixture in the fresh state was measured with the method of the ASTM standard: “Test Method for Density (Unit Weight), Yield, and Air Content (Gravimetric) of Concrete (ASTM C 138)” [26]. Then, the unit weight (kg/m^3^) of each raw material is calculated based on the mixing ratio, and the unit cost (USD/m^3^) of the mixture is determined according to the market price of each raw material. The unit price of raw materials (USD/kg) is shown in Table 3.

This study used a VEFA3 XMU SEM for microscopy experiments with an electronic image resolution of 3 nm, an acceleration voltage of 0.2~30 kV, and a magnification of 1–10000×. EDS tests can be performed at the same time. SEM was used to monitor the development of the internal structure of the CPB with an optimal mix ratio at 1 d, 3 d, 7 d, 14 d, and 28 d. First, the overall internal structural morphology of each phase was studied at low magnification (100×). The porosity was determined with ImageJ image processing software. Then, the morphology of the hydration products was observed under high magnification (5000×). EDS was used for the elemental analysis of hydration products.

## 3. Results and Discussion

### 3.1. The Results of the Response Surface Method

Based on the CCD method, a total of 30 mix ratio tests were performed (including 6 center-point repeat tests). Thirty groups of tests were performed randomly and were labelled as test numbers 1~30. The mixing ratio and response values are shown in Table 4. The code for the factor 1 aggregate-binder ratio is A, the code for the factor 2 alkali dosage is B, the code for the factor 3 solid content is C, and the code for the factor 4 air-entraining agent dosage is D. Response 1 is slump (mm), response 2 is 28-d UCS (MPa), and response 3 is cost (USD).

### 3.2. Response Surface Model Fitting and Verification

The second-order model is often used for response surface function fitting, and its form is as shown in Equation (1) [27].

(1) $$y=b_{0}+\sum_{i=1}^{k}b_{i}x_{i}+\sum_{i=1}^{k}b_{ii}x_{i}^{2}+\sum_{i<j}\sum b_{ij}x_{i}x_{j},$$

In Equation (1), *y* represents the response value, and *b_0_*, *b_i_*, *b_ii_*, and *b_ij_* represent constant coefficients, linear coefficients, quadratic term coefficients, and interaction coefficients, respectively. Here, *x_i_* and *x_j_* represent independent variables.

The slump fitting function is shown in Equation (2):

slump = −52010.8 − 1586.9*A* + 375.3*B* + 1675.2*C* − 17884.8*D* − 47.8*AB* + 15.3*AC* − 187.5*AD* − 4.3*BC* + 109.4*BD* + 284.4*CD* + 118.5*A*^2^ + 5.5*B*^2^ − 13.2*C*^2^ − 2009.4*D*^2^, (2)

The 28-d UCS fitting equation is shown in Equation (3):

28-d UCS = −81.6 − 5.9*A* − 1.8*B* + 2.5*C* + 10.3*D* + 0.3*AB* + 0.03*AC* − 2.1*AD* + 0.02*BC* + 0.2*BD* − 0.3*CD* + 0.09*A*^2^ − 0.08*B*^2^ − 0.02*C*^2^ + 27.4*D*^2^, (3)

The cost fitting function is shown in Equation (4):

cost = − 185 – 30.5*A* + 1.3*B* + 7.0*C* + 38.2*D* − 0.2*AB* + 0.2*AC* – 1.1*AD* − 5.0*BC* – 0.5*BD* – 0.5*CD* + 2.4*A*^2^ + 0.04*B*^2^ − 0.06*C*^2^ – 0.8*D*^2^, (4)

Variance analysis was performed on the above response surface functions, and the results are shown in Table 5.

Table 5 shows that the *p* values of the regression models of the slump, 28-d UCS, and cost are all <0.01, indicating that these three mathematical models are statistically significant. In addition, the critical value of F, F-tab = F_0.05_ (14, 15) = 2.42, and the calculated F values of the statistical models of the slump, 28-d UCS, and cost are 27.97, 10.08, and 32.69, respectively, which are all greater than 2.42, indicating that the statistical models of the slump, 28-d UCS, and cost are significant at a significance level of α = 0.05.

The R-squared values of the fitting equations of the slump, 28-d UCS, and cost are 0.9631, 0.9039, and 0.9683, respectively, which indicates that the three statistical models can explain the changes in response values of 96.31%, 90.39%, and 96.83%, respectively, indicating that the experimental error is not obvious.

**Figure 3:**
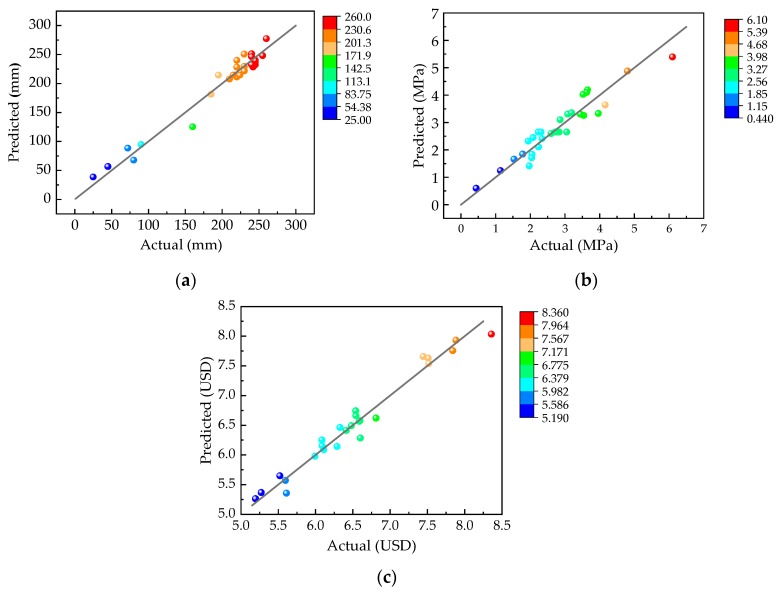
Comparison of predicted and true values: (**a**) Comparison of predicted and true values for slump; (**b**) comparison of predicted and true values for 28-d unconfined compressive strength (UCS); and (**c**) comparison of predicted and true values for cost.

To further visually show the correlation between the model and the experimental values, a comparison chart between the predicted values of the slump, 28-d UCS, and cost and their actual values is plotted, as shown in Figure 3. Figure 3 shows the scatter diagrams of the actual measurement results of 30 sets of experiments in the CCD design as the x coordinate and the response surface prediction results as the y coordinate. If all the points in the figure fall near the straight line y = x, then the correlation degree of the response surface model is high. Therefore, Figure 3 can compare the relationship between the predicted values and the experimental values.

### 3.3. Effect of Response Surface Parameters on the Slump of the CPB Mix

The analysis of variance of the slump response surface model is shown in Table 6.

The *p* values of the factor C solid content and factor D air-entraining agent dosage in Table 6 are both less than 0.01, indicating that the main effect of solid content and air-entraining agent on slump is very significant. The influence of factor C and factor D on the CPB mixture slump is discussed below.

As the solid content increases, the slump decreases. Comparing the results of test number 18 and test number 2 in Table 4, under the same test conditions, the solid content decreased from 70% to 66%, and the slump value increased from 90 mm to 220 mm with a growth rate of 144.44%. The results show that the increase in solid content reduces the slump value significantly. On the one hand, because of the increase in solid content, the distance between the aggregate particles of the CPB mixture is reduced. In the slump test, the sliding between aggregate particles is more likely to generate friction, and the fluidity deteriorates, thereby reducing the slump value [15,28]. On the other hand, an increase in solid content leads to an increase in the consistency of the CPB slurry, a decrease in fluidity, and a decrease in the slump value [29].

The influence of the air-entraining agent dosage on the slump value of the CPB mixture is shown in Table 4. The comparison of the results of test number 8 and test number 13 in Table 4 shows that under the same test conditions, the air-entraining agent dosage increased from 0 to 0.4%, and the slump value increased from 25 mm to 230 mm, with a growth rate of 820.00%. It can be seen that the increase in air-entraining agent dosage significantly increases the slump value. The reasons are as follows: First, BA is a loose, porous, sharp-edged aggregate with an extremely rough surface. The high friction between the BA particles reduces the fluidity of the CPB mixture, leading to a decrease in the slump value. In addition, BA, as a light aggregate, has a light weight, and it is difficult for it to collapse under only a high friction force with low self-gravitation. The air-entraining agent mixes the air bubbles between the aggregates. Under the support of the air bubbles, the friction between the BA particles is greatly reduced. The air bubbles act as ball bearings in the CPB slurry, which greatly reduces the surface friction between aggregate particles, and increased slurry fluidity leads to a larger slump value [30].

**Figure 4:**
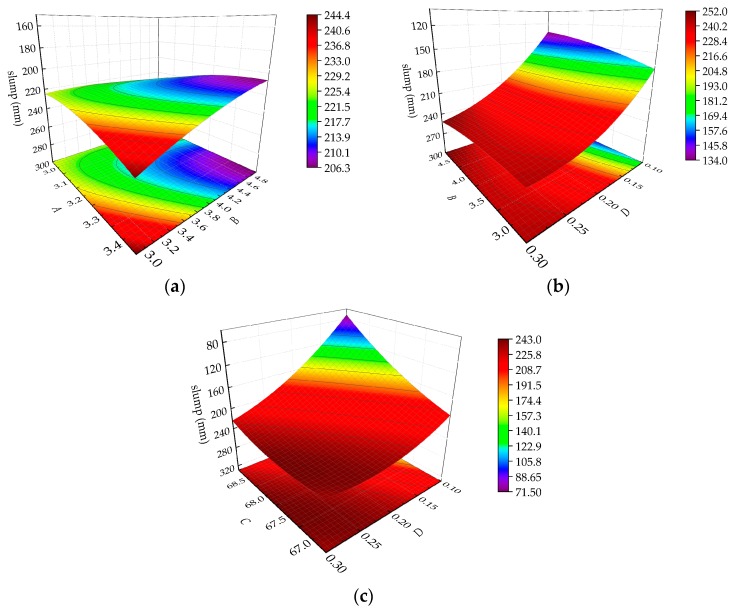
Interaction response surface diagram of various factors on the slump: (**a**) Interaction response surface diagram of AB on the slump, (**b**) interaction response surface diagram of BD on the slump, and (**c**) interaction response surface diagram of CD on the slump.

In addition, it can be seen from the analysis of variance that the *p* value of *AB* and *BD* is less than 0.05, indicating that the interaction between *AB* and *BD* is more obvious than the other interactions. The *p* value of *CD* is less than 0.0001, indicating that the interaction of CD is extremely significant. The three-dimensional surface of the response surface of the interaction of *AB*, *BD*, and *CD* is shown in Figure 4. In Figure 4, the three-dimensional response surface is clearly curved, indicating that there is a significant interaction between the factors. In Figure 4a, as A increases and B decreases, the curvature of the slump response surface (i.e., the slump growth rate) increases. The response surface of the slump in Figure 4b shows that there is a significant interaction between B and D, wherein as B decreases, D increases and the slump growth rate decreases. As shown in Figure 4c, as C decreases, D increases and the value of the slump increases, but the curvature of the surface decreases, indicating that the slump growth rate decreases.

### 3.4. Effect of Response Surface Parameters on the 28-d UCS of CPB

The analysis of variance of the response surface model of the 28-d UCS is shown in Table 7. The *p* values of the four main effects A, B, C, and D in Table 7 are all <0.01, indicating that the four main effects have a significant influence on the 28-d UCS main effect. The following section analyzes and discusses the effects of A, B, C, and D on the 28-d UCS.

The test data of test number 5 and test number 20 are compared in Table 4. Under the same test conditions, the aggregate-binder ratio changed from 2.75 to 3.75, the 28-d UCS decreased from 4.16 MPa to 2.03 MPa, and the decrease rate was 51.20%. The results show that the aggregate-binder ratio increases and that the 28-d UCS decreases. Interestingly, this result is consistent with the findings of Lee [31] et al. but is in contrast to the findings of Park [32] et al. The reason for this discrepancy is that the elemental content in the raw materials is different. In the study of Park et al. [32], the Ca content of the BA used was 52.7%, while the Ca content of the BA used in this study was only 5.3%. The BA activity is extremely low, and the active Si and Al are difficult to release under the stimulation of NaOH. However, the slag is excited by NaOH to produce a polymer that endows the slag particles with a cohesive force, wrapping the BA, filling the internal pores, and making the whole structure bind together [33]. Therefore, in this study, it is considered that the hydration products produced by alkali-activated slag are the main source of strength of hardened CPB materials. As the aggregate-binder ratio increases, the amount of binder in the CPB material decreases, and the hydration products decrease, resulting in a decrease in the compactness of the CPB material and a decrease in the CPB strength.

Comparing the 28-d UCS of test number 14 and test number 10 in Table 4, it can be seen that under the same test conditions, the alkali dosage is reduced from 6% to 2%, and the 28-d UCS is reduced from 3.54 MPa to 1.97 MPa, with a decrease rate of 44.35%. The reasons for this result may be that NaOH is an activator of alkali-activated slag binder. The increase in alkali dosage causes more activated alumina and silica leaching and generates more hydration products through polycondensation. The hydration products connect the slag particles and BA particles, which fill the pores, resulting in an increase in the 28-d UCS [15,34].

Comparing the 28-d UCS of test number 18 and test number 2 in Table 4, under the same test conditions, when the solid content is reduced from 70% to 66%, the 28-d UCS is reduced from 3.96 MPa to 2.05 MPa, and the increase rate is 48.23%. It shows that the solid content is reduced, and the 28-d UCS is significantly reduced. The reason is that the increase in solid content is equivalent to the decrease in water content. On the one hand, the initial porosity of the CPB was reduced, and the alkali-activated slag binder matrix was more tightly bonded to the BA particles. On the other hand, the reduction of water content indirectly increases the alkali concentration, and the alkaline environment promotes the hydration process of slag.

The 28-d UCS values of test number 8 and test number 13 are compared in Table 4. Under the same test conditions, the air-entraining agent dosage was increased from 0% to 0.4%, and the 28-d UCS decreased from 6.1 MPa to 2.24 MPa, with a reduction rate of 63.28%. The results showed that the increase in air-entraining agent dosage reduced the 28-d UCS value. The reason for this result may be that the incorporation of air-entraining agents introduces holes in the matrix of the original average gel material. Microcracks are formed around the holes. Under the load, stress concentration occurs, which causes the structure to easily break and reduces the strength [30].

From the analysis of variance of the 28-d UCS response surface model in Table 7, the *p* value of each interaction term is greater than 0.05, showing that the influence of each interaction term on the 28-d UCS is not obvious, so they are not analyzed here. However, the addition of a quadratic term and the interaction term increases the correlation coefficient of the fitting equation, so this study still uses the second-order function with the interaction term as the 28-d UCS response surface regression model.

### 3.5. Effect of Response Surface Parameters on the Cost of CPB

The analysis of variance of the cost response surface model is shown in Table 8. Among the *p* values, the *p* values of factors A, B, and D are all less than 0.01, which indicates that the effects of A, B, and D on the cost of the CPB are extremely significant. The following is an analysis of the impact of A, B, and D on cost.

The cost values of test number 5 and test number 20 are compared in Table 4. Under the same test conditions, the aggregate-binder ratio changes from 2.75 to 3.75, the cost decreases from 8.36 USD to 5.99 USD, and the reduction rate is 28.35%. This result shows that the larger the aggregate-binder ratio is, the lower the cost. The reason is that when the alkali dosage, solid content, and air-entraining agent dosage are the same, the aggregate-binder ratio increases, the amount of slag per unit volume increases, and the cost increases.

The costs of test number 14 and test number 10 are compared in Table 4. Under the same test conditions, the alkali dosage is reduced from 6% to 2%, the cost is reduced from 7.84 USD to 5.61 USD, and the reduction rate is 28.44%. This shows that the alkali dosage is reduced, and the cost is significantly reduced. The reason may be that in the alkali-activated slag CPB material, under the same aggregate-binder ratio, solid content, and air-entraining agent dosage, the adjustment of the alkali dosage affects the apparent density of the material and is close to zero. However, the more NaOH the CPB mix dissolves, the higher its cost.

Comparing the cost values of test number 13 and test number 8 in Table 4, it can be seen that under the same test conditions, the air-entraining agent dosage is reduced from 0.4% to 0%, and the cost is reduced from 6.81 USD to 6.29 USD with a reduction rate of 7.64%. This shows that the air-entraining agent dosage is reduced, and the cost is reduced. Even if the dosage of the air-entraining agent is increased, the apparent density of the CPB mixture is reduced, that is, the quality of the raw materials used per unit volume is reduced. However, the unit price of the air-entraining agent is much higher than the unit prices of the other raw materials (see Table 3 for the unit prices of the raw materials). Therefore, the unit price of the air-entraining agent dominates the impact of the cost of materials. Therefore, the dosage of the air-entraining agent increases, and the cost of CPB materials increases.

Table 8 shows the analysis of variance of the response surface model of cost; the *p* value of each interaction term is greater than 0.05, showing that the influence of each interaction item on the cost is not obvious, so they are not analyzed here. The addition to the interaction term increases the correlation coefficient of the fitting equation, this study still uses the second-order function with the interaction term as the response surface regression model of cost.

### 3.6. Multi-Objective Optimization

This paper refers to the desirability function method in [35] to deal with the multi-objective optimization problem to obtain the optimal mix ratio of CPB.

The general idea of multi-objective optimization is as follows: First, establish the single desirability function *d_i_* of every response according to the type (“maximum,” “minimum,” and “target”). Then, according to the experimental results of the CCD design, the lower limit, upper limit, and response surface equations of each response, namely, *Low_i_*, *High_i_*, and *Y_i_*, are brought into the desirability function of a single response. The overall desirability function, *D*, is the geometric mean of all single desirability functions. Nonlinear programming is performed on *D*. When *D* obtains the maximum value, the parameter value of each single satisfaction function is the optimal mixture ratio.

The principles of CPB optimization in this study are as follows: the slump reaches the target value of 200 mm, the 28-d UCS is maximized, and the cost is minimized. That is, under the condition that the CPB slurry working performance is satisfactory, the strength of the backfill is maximized, and the cost of the CPB material per cubic meter is minimized. Therefore, the type of slump is “target,” the type of 28d-UCS is “maximum,” and the type of cost is “minimum.”

First, a single response satisfaction function *d_i_* based on a response surface model of the slump, 28-d UCS, and cost is established.

For the single response, the satisfaction function *d_i_* of the slump is a function for the goal as a target and should be calculated according to Equation (5) [35].

(5) $$\begin{array}{l} d_{i}=\{\begin{array}{cc} 0, & Y_{i}\leq Low_{i}\\ {\left[\frac{Y_{i}-Low_{i}}{T_{i}-Low_{i}}\right]}^{wt_{i}}, & Low_{i}<Y_{i}<T_{i}\\ 1, & Y_{i}=T_{i}\\ {\left[\frac{High_{i}-Y_{i}}{High_{i}-T_{i}}\right]}^{wt_{i}}, & T_{i}<Y_{i}<High_{i}\\ 0, & Y_{i}\geq High_{i} \end{array} \end{array}$$

where *d_i_* represents the satisfaction function of the i-th response surface, which is the satisfaction function of the slump. *Y_i_* is the i-th response, which is the response surface function for the slump. *Low_i_* is the lower limit of the i-th response value, which is the minimum value of the slump test result in the CCD design (25mm). *High_i_* is the upper limit of the i-th response value, which is the maximum value of the slump test result in the CCD design (260mm). *T_i_* is the target value of the i-th response surface, and the target value of the slump in this study is 200 mm. *wt_i_* represents the weight factor of the i-th satisfaction function, wherein 0.1 ≤ *wt_i_* ≤ 10. The weight factor *wt_i_* can change the shape of the satisfaction function. When *wt_i_* is equal to 1, *d_i_* changes from 0 to 1 in a linear form. If *wt_i_* is less than 1, the degree of emphasis on the target is low, *d_i_* is a convex function, and the rate of change from 0 to 1 gradually slows. If *wt_i_* is greater than 1, the degree of emphasis on the target is higher, the *d_i_* function is concave, and the rate of change from 0 to 1 gradually increases. In this study, the weight factor *wt_i_* = 1 is selected.

Similarly, *d_i_* represents the satisfaction function of 28d-UCS. *Y_i_* is the response surface function for 28d-UCS. *Low_i_* is the minimum value of the 28d-UCS test result in the CCD design, which is 0.44MPa. *High_i_* is the maximum value of the slump test result in the CCD design, which is 6.10MPa; the weight factor *wt_i_* is equal to 1.

The 28-d UCS single response satisfaction function is a function for the goal as a maximum and should be calculated according to Equation (6) [35].

(6) $$d_{i}=\{\begin{array}{cc} 0, & Y_{i}\leq Low_{i}\\ {\left[\frac{Y_{i}-Low_{i}}{High_{i}-Low_{i}}\right]}^{wt_{i}}, & Low_{i}<Y_{i}<High_{i}\\ 1 & Y_{i}\geq High_{i} \end{array},$$

The cost single response satisfaction function is a function for the goal as a minimum, which should be calculated according to Equation (7) [35].

(7) $$d_{i}=\{\begin{array}{cc} 1, & Y_{i}\leq Low_{i}\\ {\left[\frac{H{\mathrm{igh}}_{i}-Y_{i}}{High_{i}-Low_{i}}\right]}^{wt_{i}}, & Low_{i}<Y_{i}<High_{i}\\ 0 & Y_{i}\geq High_{i} \end{array},$$

Similarly, *d_i_* represents the satisfaction function of cost. *Y_i_* is the response surface function for cost. *Low_i_* is the minimum value of the cost in the CCD design, which is 5.20 USD. *High_i_* is the maximum value of the cost in the CCD design, which is 8.36 USD; and the weight factor *wt_i_* is equal to 1.

Finally, an overall satisfaction function (i.e., desirability function) *D* is established, which is equal to the geometric mean of the desired goals *d_i_* of all responding individuals [35], which is

(8) $$D={\left(d_{1}^{r_{1}}\times d_{2}^{r_{2}}\times d_{3}^{r_{3}}\right)}^{\frac{1}{\sum r_{i}}},$$

where *r_i_* represents the importance degree of each response, $\sum r_{i}=1$, and the greater *r_i_* the more important it is. This study refers to [36] and considers that the slump, 28-d UCS, and cost are equally important, that is, *r*_1_ = *r*_2_ = *r_3_* *= 1/3*. With the above single response satisfaction functions as the constraint conditions, nonlinear programming is performed on *D* to obtain a set of mixture ratios, and the one with the highest *D* value is selected as the optimal mixture ratio.

Through optimization analysis, the maximum point of optimization result D is found. At this time, the aggregate-binder ratio was 3.28, the alkali dosage was 3.00%, the solid content was 67.44%, and the air-entraining agent dosage was 0.10%. The predicted value of slump is 200 mm, the predicted value of 28-d UCS is 2.94 MPa, and the predicted cost is 5.59 USD. Table 9 compares the experimental and predicted values of the optimized mixture.

Under the same test conditions, the CPB is configured with the optimal mix ratio. The measured slump is 205 mm, the 28-d UCS is 2.93 MPa, and the cost is 5.70 USD. The absolute relative deviation (*ARD*) of the predicted and experimental values is calculated according to the following formula [37].

(9) $$ARD(\%)=\frac{Exprimental-Predicted}{Experimental}\times 100,$$

The *ARDs* for slump, 28-d UCS, and cost were 2.44%, −0.34%, and 2.00%, respectively.

**Figure 5:**
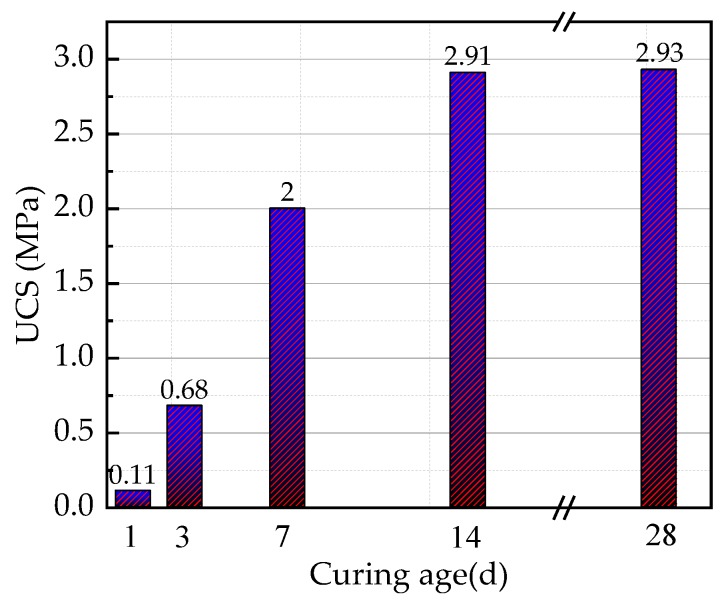
Diagram for the changes in the UCS of cemented paste backfill (CPB) with curing age.

In addition, the UCS of CPB changes with curing age, as shown in Figure 5. With an increasing age of 1 d, 3 d, 7 d, 14 d, and 28 d, the UCS values were 0.11 MPa, 0.68 MPa, 2.00 MPa, 2.91 MPa, and 2.93 MPa, respectively.

### 3.7. Microstructural Analysis

To explain the strength development mechanism of CPB with curing age under the optimal mixing ratio, the micro-characterization method of SEM-EDS was used to determine the internal microstructure of CPB. The evolution of the macromechanics is explained from two aspects: the change rate of microscopic pores and the change in hydration reaction products.

#### 3.7.1. Changes in Porosity with Curing Age

**Figure 6:**
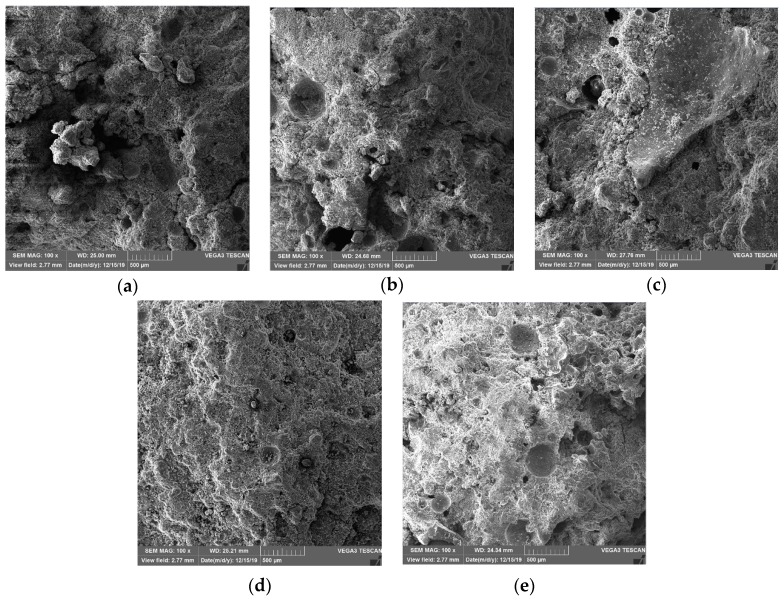
The scanning electron microscopy (SEM) micromorphology of samples at different curing periods at 100× magnification: (**a**) 1 d, (**b**) 3 d, (**c**) 7 d, (**d**) 14 d, and (**e**) 28 d.

**Figure 7:**
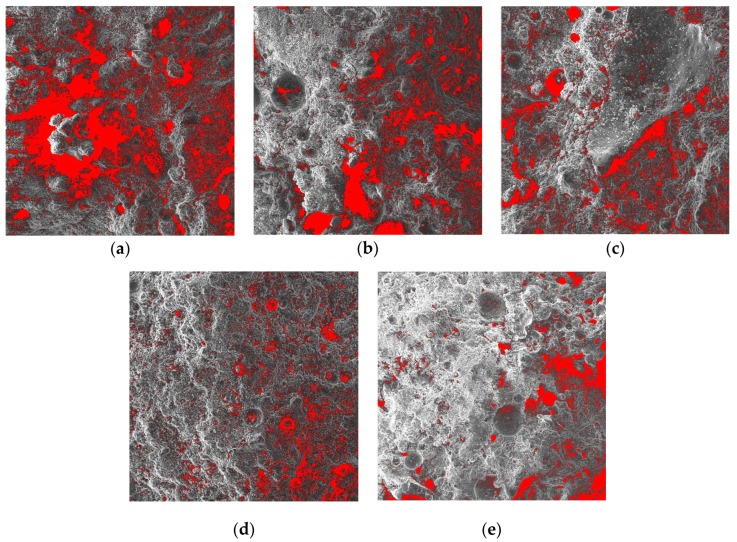
Pore structure analysis at different curing periods: (**a**) 1 d of pore structure analysis, (**b**) 3 d of pore structure analysis, (**c**) 7 d of pore structure analysis, (**d**) 14 d of pore structure analysis, and (**e**) 28 d of pore structure analysis.

SEM was used to magnify the 1-d, 3-d, 7-d, 14-d, and 28-d CPB samples 100 times, as shown in Figure 6. From the SEM images, it can be seen that as the age increases, the internal microstructure of the CPB sample becomes denser. To further detect the pore changes, the image processing software ImageJ was used to process a 100-fold magnified SEM image, as shown in Figure 7. The porosities from 1 d, 3 d, 7 d, 14 d, and 28 d were 23.42%, 12.82%, 9.87%, 7.25%, and 6.79%, respectively. This shows that as the age increases, the internal structure of CPB becomes dense.

#### 3.7.2. Microstructural Changes and Hydration Product Types Increase with Curing Age

**Figure 8:**
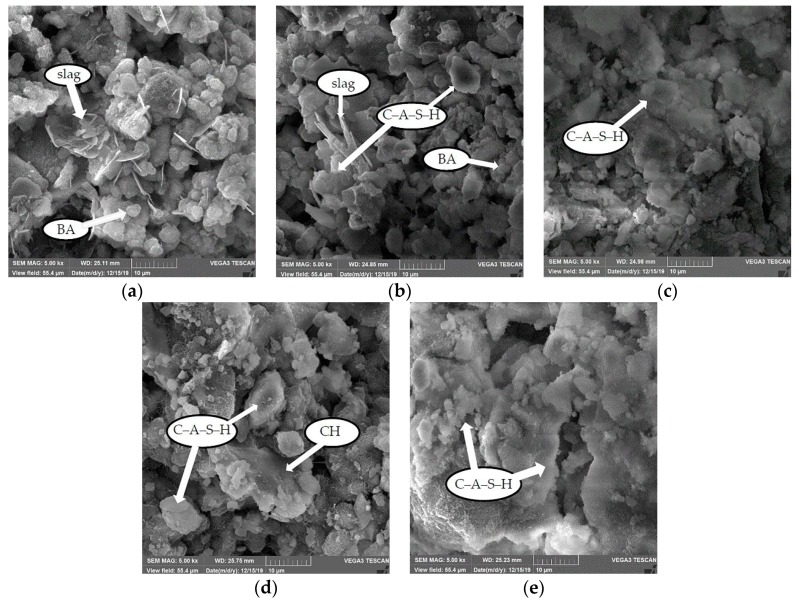
SEM micromorphology of samples at different curing ages (5000× magnification): (**a**) 1 d, (**b**) 3 d, (**c) 7** d, (**d**) 14 d, and (**e**) 28 d.

The samples at 1 d, 3 d, 7 d, 14 d, and 28 d were enlarged 5000 times by SEM, as shown in Figure 8a–e. With the progress of CPB polycondensation, the microstructures at different ages are different. In general, the microstructure consists of two parts: a cemented phase and a residual phase. The cemented phase plays a leading role, and its type, relative size, and spatial distribution determine the strength of the CPB material.

**Figure 9:**
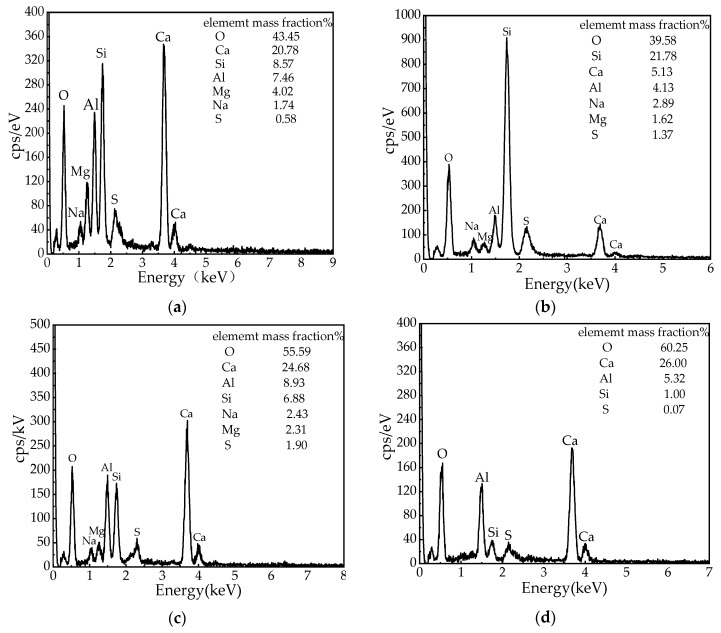
Energy dispersive spectroscopy (EDS) energy spectrum analysis: (**a**) EDS analysis of slag, (**b**) EDS analysis of BA, (**c**) EDS analysis of C-A-S-H, (**d**) EDS analysis of C-H.

Figure 8a shows the micromorphology of CPB hydration at 1 d. It can be seen from Figure 8a that at 1 d, irregular plate-like, small spherical and small flocculent substances appear inside the CPB. Small spherical materials fill the entire space uniformly, irregular plate-like materials are randomly scattered inside the CPB, which is partly covered with small spherical materials, and a small amount of flocculent materials are wrapped around the small spherical materials and are connected with the plate-like materials. The microstructure determined by SEM combined with EDS was used to determine the type of hydration product. For irregular plate-like substances, from the perspective of micromorphology, slag presents an irregular plate-like microstructure [30]. Further analysis by EDS (Figure 9a) shows that the main elements of the irregular plate-like substance are Ca, Si, and Al, which is in good agreement with the chemical element ratio of the slag, identified as unreacted slag. The EDS spectrum of the small spherical materials is shown in Figure 9b. Si has the largest proportion, and Ca, Al, and the other elements are present in relatively small proportions. Compared with the XRF of the raw materials, the small spherical substance is BA. Flocculent matter appears at all ages and is the main cemented phase. With increasing age, flocculent substances develop continuously, and the degree of crystallization increases. However, the micromorphological characteristics are consistent with the element content characteristics of the EDS spectrum. The EDS spectrum of a typical flocculent substance is shown in Figure 9c. Ca, Si, and Al coexist in large quantities, and it was inferred that the flocculent substance was a calcium aluminosilicate hydrate (C-A-S-H) gel because the Al content in the slag is large. In the calcium silicate hydrate (C-S-H) gel formed by the slag reaction, Al partially replaces Si in the C-S-H gel, thereby forming a C-A-S-H gel [38].

Figure 8b shows the microscopic morphology of CPB hydration for 3 d. Compared with the 1 d sample, the small spherical substance, BA, inside the CPB was greatly reduced because of the tight encapsulation of C-A-S-H at 3 d; the amount of unreacted slag decreased, staggered with C-A-S-H, and the amorphous floc was further expanded into agglomerated bulbous floc. That is, the unreacted slag gradually dissolves and is reduced, and more C-A-S-H gels are generated, while BA is reduced because it is coated with C-A-S-H gels.

Figure 8c shows the micromorphology of CPB hydration at 7 d. Compared with the previous period, there is almost no irregular plate-like substance in the test sample at 7 d; that is, the slag is completely dissolved. The small spherical BA is tightly covered by flocs, and the mass of C-A-S-H expands outward to fill the overall structure more tightly.

Figure 8d shows the micromorphology of CPB hydration at 14 d. It can be seen from a relatively complete, large crystal hexagonal plate-like substance is embedded in the floc C-A-S-H in the microstructure of CPB. For the hexagonal plate-like material with large crystals, the EDS spectrum is shown in Figure 9d, revealing that the material contains a large amount of Ca, and the remaining elements are less abundant than Ca; it is inferred that calcium hydroxide (CH) is present. Xu et al. [39] described that the degree of crystallization of portlandite is relatively complete, and the crystals are relatively large and are mainly hexagonal plate-shaped, layered, or plate-like substances, which is consistent with the conclusions of this study.

Figure 8e shows the micromorphology of CPB hydration at 28 d. The flocculent C-A-S-H gel in the sample is connected to a whole structure, and the cementation is tight. It has been confirmed in the literature [40,41,42] that aluminosilicate hydrate is an important source of high-strength alkali-activated materials. The degree of hydration and crystallization of the hydration products is extremely high, and the crystal grains are significantly increased.

## 4. Conclusions

(1) Based on the CCD response surface method, mathematical models of the CPB material slump, 28-d UCS, and cost are established. The optimal mix ratio with an aggregate-binder ratio of 3.28, an alkali dosage of 3.00, a solid content of 67.44%, and an air-entraining agent dosage of 0.10% was obtained by the desirability function method. The measured slump is 205 mm, the 28-d UCS is 2.93 MPa, and the cost is 5.70 USD /m^3^.
(2) The microanalysis of the optimal mix ratio shows that as the curing age increases, the internal porosity of CPB gradually decreases. The hydration products of alkali-activated slag are mainly C-A-S-H gels. At the beginning of the hydration reaction, as the age increases, slag is gradually consumed, and almost all the slag participates in the reaction at 7 d. With the progress of the polycondensation reaction, the C-A-S-H gel continuously wraps the BA surface, thereby increasing the strength.
(3) At 14 d, CHwith a high degree of crystallization and a relatively complete morphology is embedded in the hydration products. At 28 d, the C-A-S-H flocs are connected as a whole, forming a dense structure with a high degree of hydration. The formation of microscopic hydration products and the denseness of the pore structure cause the CPB strength to increase with age.

## Figures and Tables

**Table 1:** Main chemical composition of raw materials (wt%).

Materials	SiO_2_	Al_2_O_3_	CaO	Fe_2_O_3_	MgO	SO_3_	Na_2_O
BA	56.7	18.9	5.3	10.3	1.9	0.5	1.1
Slag [23]	27.5	16.2	40.8	0.5	7.8	2.9	0.3

**Table 2:** Factors, numbers, levels, and units of the mix ratio design.

Factors	Code	Unit	Level				
			−2	−1	0	1	2
Aggregate-binder ratio	*A*	-	2.75	3	3.25	3.5	3.75
Alkali dosage	*B*	%	2	3	4	5	6
Solid content	*C*	%	66	67	68	69	70
Air-entraining agent dosage	*D*	%	0	0.1	0.2	0.3	0.4

**Table 3:** The unit price of raw materials.

Materials	Slag	NaOH	H_2_O	Air-Entraining Agent
Unit Price (USD/kg)	0.0137	0.289	0.000665	1.44

**Table 4:** Central composite design (CCD) test results.

Test Number	Factor 1	Factor 2	Factor 3	Factor 4	Response 1	Response 2	Response 3
	*A*	*B*	*C*	*D*	Slump	28-d UCS	Cost
		%	%	%	mm	MPa	USD
1	3	5	69	0.3	240	3.08	7.51
2	3.25	4	66	0.2	220	2.05	6.60
3	3	3	69	0.3	242	2.08	6.33
4	3.5	5	69	0.1	45	3.65	6.60
5	2.75	4	68	0.2	255	4.16	8.36
6	3.25	4	68	0.2	220	3.05	6.41
7	3.25	4	68	0.2	218	2.83	6.41
8	3.25	4	68	0	25	6.10	6.29
9	3	5	67	0.3	260	2.60	7.88
10	3.25	2	68	0.2	230	1.97	5.61
11	3.5	3	69	0.1	160	2.86	5.27
12	3.5	5	67	0.3	245	1.53	6.54
13	3.25	4	68	0.4	230	2.24	6.81
14	3.25	6	68	0.2	230	3.54	7.84
15	3	3	67	0.1	220	3.19	6.09
16	3	3	67	0.3	245	1.78	6.54
17	3.25	4	68	0.2	215	2.78	6.41
18	3.25	4	70	0.2	90	3.96	6.11
19	3.5	3	67	0.3	245	0.44	5.52
20	3.75	4	68	0.2	220	2.03	5.99
21	3.25	4	68	0.2	215	2.72	6.41
22	3.5	3	69	0.3	240	1.14	5.60
23	3.5	5	67	0.1	185	3.44	6.48
24	3	5	69	0.1	80	4.80	7.52
25	3	3	69	0.1	72	3.62	6.09
26	3.25	4	68	0.2	224	2.23	6.41
27	3	5	67	0.1	210	3.52	7.44
28	3.5	3	67	0.1	240	1.94	5.20
29	3.5	5	69	0.3	230	2.34	6.59
30	3.25	4	68	0.2	195	2.31	6.41

**Table 5:** Model validation for the response.

Response	Slump	28-d UCS	Cost
Degree of freedom			
Regression	14	14	14
Residual error	15	15	15
Standard deviation	17.55	0.48	0.19
R-Squared	0.9631	0.9039	0.9683
Adj R-Squared	0.9287	0.8142	0.9386
F value	27.97	10.08	32.69
*p* value	<0.001	<0.0001	<0.0001
Significance	Yes	Yes	Yes

**Table 6:** Analysis of variance of the slump regression model.

Variation Source	Sum of Squares	df	Mean Square	F Value	P Value
Model	120,600	14	8613.00	27.97	<0.0001
*A*	100.04	1	100.04	0.32	0.5771
*B*	1190.04	1	1190.04	3.86	0.0681
*C*	26,733.37	1	26,733.37	86.82	<0.0001
*D*	54,626.04	1	54,626.04	177.41	<0.0001
*AB*	2280.06	1	2280.06	7.40	0.0158
*AC*	232.56	1	232.56	0.76	0.3985
*AD*	351.56	1	351.56	1.14	0.3022
*BC*	297.56	1	297.56	0.97	0.3412
*BD*	1914.06	1	1914.06	6.22	0.0248
*CD*	12,939.06	1	12,939.06	42.02	<0.0001
*A^2^*	1504.53	1	1504.53	4.89	0.043
*B^2^*	839.17	1	839.17	2.73	0.1195
*C^2^*	4792.74	1	4792.74	15.57	0.0013
*D^2^*	11,074.53	1	11,074.53	35.97	<0.0001
Residual	4618.75	15	307.92	-	-
Lack of Fit	4105.25	10	419.52	4.00	0.0697
Pure Error	513.50	5	102.70	-	-
Cor Total	125,200	29	-	-	-

**Table 7:** Analysis of variance of the 28-d UCS regression model.

Variation Source	Sum of Squares	df	Mean Square	F Value	*p* Value
Model	32.97	14	2.35	10.08	<0.0001
A	5.60	1	5.60	23.95	0.0002
B	5.09	1	5.09	21.77	0.0003
C	3.34	1	3.34	14.28	0.0018
D	16.25	1	16.25	69.56	<0.0001
AB	0.098	1	0.098	0.42	0.5277
AC	0.00141	1	0.00141	0.0062	0.9392
AD	0.045	1	0.045	0.19	0.6665
BC	0.012	1	0.012	0.049	0.827
BD	0.00601	1	0.00601	0.026	0.8748
CD	0.019	1	0.019	0.081	0.78
A^2^	0.000774	1	0.000774	0.000774	0.9549
B^2^	0.17	1	0.17	0.75	0.4015
C^2^	0.0081	1	0.0081	0.035	0.8548
D^2^	2.06	1	2.06	8.82	0.0096
Residual	350	15	0.23	-	-
Lack of Fit	3.00	10	0.30	2.96	0.1211
Pure Error	0.51	5	0.10	-	-
Cor Total	36.47	29	-	-	-

**Table 8:** Analysis of variance for cost regression models.

Variation Source	Sum of Squares	df	Mean Square	F Value	P Value
Model	16.34	14	1.17	32.69	<0.0001
*A*	6.34	1	6.34	177.57	<0.0001
*B*	8.63	1	8.63	241.74	<0.0001
*C*	0.058	1	0.058	1.61	0.2238
*D*	0.34	1	0.34	9.60	0.0073
*AB*	0.03	1	0.03	0.85	0.3704
*AC*	0.042	1	0.042	1.17	0.2972
*AD*	0.011	1	0.011	0.31	0.5862
*BC*	0.0004	1	0.0004	0.011	0.9177
*BD*	0.047	1	0.047	1.31	0.2701
*CD*	0.034	1	0.034	0.94	0.3478
*A^2^*	0.61	1	0.61	16.99	0.0009
*B^2^*	0.036	1	0.036	1.01	0.3316
*C^2^*	0.086	1	0.086	2.42	0.1408
*D^2^*	0.0015	1	0.0015	0.043	0.8379
Residual	0.54	15	0.54		
Lack of Fit	0.54	10	0.54		
Pure Error	0.00	5	0.00		
Cor Total	16.88	29			

**Table 9:** Comparison between the experimental and predicted values of the optimized mixture.

Term	Slump(mm)	28d-UCS(MPa)	Cost(USD)
Experimental	205	2.93	5.70
Predicted	200	2.94	5.59
